# Screening of Carbofuran-Degrading Bacteria *Chryseobacterium* sp. BSC2-3 and Unveiling the Change in Metabolome during Carbofuran Degradation

**DOI:** 10.3390/metabo12030219

**Published:** 2022-03-01

**Authors:** Haeseong Park, Sun Il Seo, Ji-Hwan Lim, Jaekyeong Song, Joo-Hyun Seo, Pyoung Il Kim

**Affiliations:** 1 Center for Industrialization of Agricultural and Livestock Microorganisms, 241 Cheomdangwahak-ro, Jeongeup-si 56212, Korea; haeseong@cialm.or.kr (H.P.); siseo@cialm.or.kr (S.I.S.); ljh0408@cialm.or.kr (J.-H.L.); 2 Division of Agricultural Microbiology, National Academy of Agricultural Science, 166 Nongsaengmyeong-ro, Wanju-gun 55365, Korea; mgjksong@korea.kr; 3 Department of Bio and Fermentation Convergence Technology, Kookmin University, Seoul 02707, Korea

**Keywords:** carbofuran, bioremediation, metabolomics, aromatic compound degradation, plant growth-promoting microorganism

## Abstract

Carbofuran is one of the most commonly used N-methylcarbamate-based pesticides and is excellent for controlling pests; however, carbofuran also causes soil and water pollution. Although various studies have been conducted on the bioremediation of pesticide-contaminated soil, the changes occurring in the metabolome during the bioremediation of carbofuran are not fully understood. In this study, the intracellular and extracellular metabolites of the *Chryseobacterium* sp. BSC2-3 strain were analysed during carbofuran degradation by using a liquid chromatography–mass spectrometry-based metabolomics approach. We found that the BSC2-3 strain extracellularly transformed carbofuran into 3-hydroxycarbofuran. Intracellular metabolite analysis revealed that carbofuran mainly affected aminobenzoate degradation, ubiquinone and terpenoid-quinone biosynthesis, and arginine and proline metabolism. Carbofuran especially affected the metabolic pathway for the degradation of naphthalene and aminobenzoate. Metabolomics additionally revealed that the strain produces disease resistance inducers and plant growth regulators. We also identified the genes involved in the production of indole-3-acetic acid, which is one of the most active auxins. Overall, we identified the metabolic changes induced in carbofuran-degrading bacteria and the genes predicted to be responsible for the degradation of carbofuran.

## 1. Introduction

Pesticides have been used to control pests in agriculture and have contributed to human society by greatly enhancing agricultural outcomes [1,2,3]. A number of pesticides have been used widely and with great effectiveness [4]. Among the various insecticides, carbofuran (2,3-dihydro-2,2-dimethyl-7-benzofuranoyl-N-methylcarbamate, C_12_H_15_NO_3_, CAS registry no. 1563-66-2), a member of the carbamate insecticide group, is a highly effective insecticide and is extensively used on cabbages, potatoes, soybeans, tomatoes and many other crops [5]. Carbofuran has become one of the dominant insecticides used in agriculture due to its ability to control a wide range of pests [6]. However, carbofuran is known as a powerful inhibitor of acetyl-cholinesterase and possesses serious toxicity, producing negative health effects for wild animals and humans and poisoning rivers and groundwater [7,8,9]. In addition, the application of carbofuran to crops results in changes in the metabolism of plants and changes the soil microbiota and their metabolites [10], which ultimately affects crop quality [11,12,13,14]. Due to its potential negative effects, the usage of carbofuran requires thorough monitoring, and its degradation from the environment has become an important research topic. Various methods have been introduced for the elimination of carbofuran in the laboratory environment, including Fenton degradation, photodegradation and oxidation by ozone [15,16,17]. However, these approaches for controlling or alleviating the ecological toxicity of chemical pesticides are ineffective, impracticable and expensive [18]. Bioremediation is considered to be an ecologically friendly and energy-efficient approach with great capacity for degrading various pesticides [19]. It has already been reported that many microorganisms degrade pesticides and use them as nutrients for growth [20]. The genera *Pseudomonas* [21] and *Achromobacter* [22] are representative microbes that are capable of degrading carbamate insecticides, including carbofuran, in various environments. Therefore, microbial remediation, instead of physical and chemical methods for pesticide removal, can be a great way to reduce the cost of soil remediation in an environmentally friendly way [23]. Several other microbes, such as *Cupriavidus* sp. [24], *Enterobacter* sp. [25], *Sphingomonas* sp. [26] and some fungal strains [27], induce efficient carbofuran biodegradation. However, most studies have focused on the isolation of carbofuran-degrading bacteria and on the expression of a few target genes. This limited information seemed to be insufficient and led us to carry out an omics study. Metabolomics is a promising field in agriculture, and non-targeted metabolomics is used to identify the changes in specific metabolic pathways caused by exogenous chemicals, which facilitates the identification of the responsible metabolic pathways involved in the utilization or degradation of exogenous chemicals. Metabolomics also helps when identifying small changes at the biomolecular level and discovering the mechanisms of intracellular metabolic processes [28]. Through an analysis of highly altered metabolites and their associated intracellular metabolism, metabolomics can provide insights into toxic insecticides and xenobiotics [29]. Therefore, in this study, we sought to isolate carbofuran-degrading bacteria and undertake an organism-wide identification of carbofuran degradation pathways by observing changes in metabolomic profiles with non-targeted metabolomics and identifying relevant genes through the genome sequencing of newly isolated bacteria.

## 2. Results and Discussion

### 2.1. Isolation of Carbofuran-Degrading Bacteria from the Enrichment Culture

**Figure 1:**
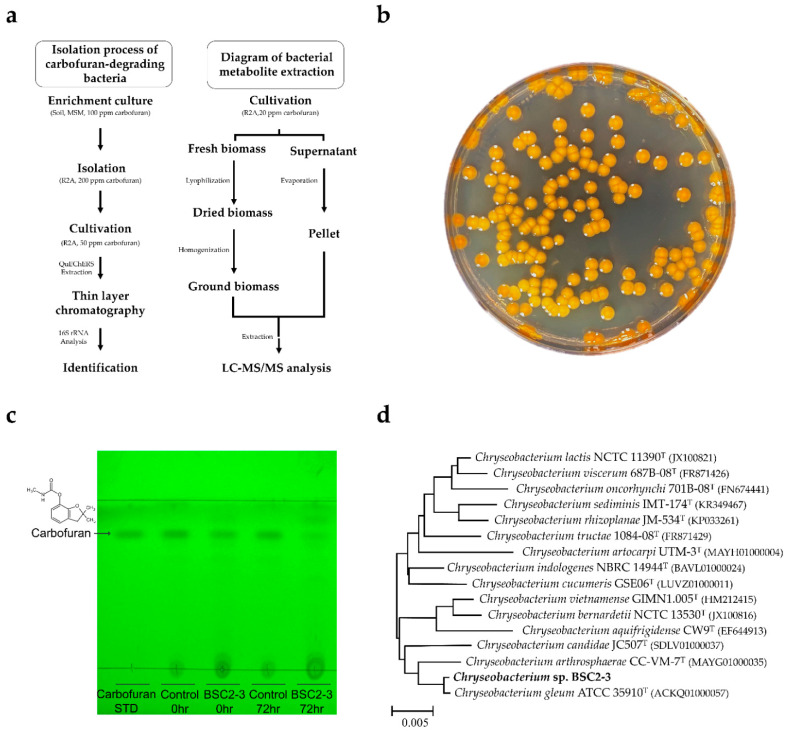
Experimental procedure, cell morphology, thin-layer chromatography results and phylogenetic tree analysis of BSC2-3 cells. (**a**) A schematic diagram of the experimental process. (**b**) BSC2-3 on R2A solid medium containing 200 ppm carbofuran. (**c**) TLC analyses of metabolites of *Chryseobacterium* sp. BSC2-3 cultured in R2A medium with 50 ppm carbofuran. Carbofuran STD, 2000 ppm carbofuran; Control 72 h, 72 h culture extract of R2A with 50 ppm carbofuran; BSC2-3 0 h, 0 h culture extract of the BSC2-3 with 50 ppm carbofuran; BSC2-3 72 h, 72 h culture extract of the BSC2-3 strain with 50 ppm carbofuran. (**d**) Phylogenetic tree of BSC2-3 based on 16S rRNA analysis. The sequences were aligned using ClustalW, which was operated by the MEGA X alignment platform with 1000 bootstrap replicates. The phylogenetic tree was constructed by using the neighbour-joining method. The scale bar represents the number of substitutions per site.

Five soil samples were prepared from an agricultural field that had been contaminated with various pesticides and insecticides for years, and a minimal salt medium (MSM) without carbon, nitrogen and sulfur sources was used for the enrichment culture. The overall experimental workflow is presented in Figure 1a. Carbofuran was added as the sole carbon source when performing enrichment culture using the minimal salt medium. Although carbofuran structurally does not contain sulfur, sufficient microbial growth was observed in the enrichment culture. Based on this result, it is expected that some carbofuran-degrading microorganisms could be grown using the sulfur obtained from organic matter or organisms present in the soil during the enrichment culture process. Among the five soil samples used for the enrichment culture, the sample in which the growth of the bacteria was confirmed in the third subculture medium was diluted and spread on an MSM plate, to which 100 ppm carbofuran was added. Among diverse strains, morphologically distinctive strains that form smooth, circular, orange-pigmented colonies with the largest colony-forming strain (BSC2-3) were selected for further study (Figure 1b).

### 2.2. Confirmation of the Carbofuran-Biodegrading Ability of BSC2-3

The degradation of carbofuran by BSC2-3 was assessed by monitoring the diminution of carbofuran spots on thin-layer chromatography (TLC) plates. Generally, individual cultures of pesticide-degrading strains are carried out in MSM [24,30,31]. For carbofuran degradation, R2A broth was employed as a culture medium, given that it better supports the survival of bacteria and promotes a more rapid cellular growth of bacteria than MSM. The BSC2-3 strain was cultured in an R2A medium containing 50 ppm carbofuran. Subsequently, the time course culture medium sample was extracted by the QuEChERS-EN method, and extracts were analysed using TLC. For visualization, TLC plates were exposed to 254 nm ultraviolet (UV) light to determine carbofuran levels and identify additional spots. As shown in Figure 1c, the carbofuran spots of the 72 h control culture extracts remained black, which indicates that carbofuran was still not degraded. On the other hand, the carbofuran spots of the BSC2-3 culture extracts faded in a time-dependent manner. The carbofuran spot of the carbofuran-treated group became clear even after 72 h of incubation. According to previous research, the degradants of carbofuran were more polar and their *R_f_* values were larger than those of carbofuran when developed in polar solvent mixtures [32]. However, spots for carbofuran degradation products were not seen in the TLC of the BSC2-3 culture extract under 254 nm UV light (Figure 1c). Therefore, it was expected that the BSC2-3 strain had the ability to degrade carbofuran into smaller molecules, which were barely visible on the TLC plate. It was also assumed that the concentration of carbofuran degradation products was not sufficient for visualization. For this reason, there is a limitation to the use of TLC analysis for identifying the carbofuran degradation products of BSC2-3.

### 2.3. Identification of Carbofuran-Degrading Bacteria

Sequence analysis of the 16S rRNA gene showed that the 16S rRNA coincidence with *Chryseobacterium gleum* was over 99.9%. *Chryseobacterium indologenes* (98.89% similarity), *Chryseobacterium lactis* (98.82%), *Chryseobacterium cucumeris* (98.54%) and *Chryseobacterium vietnamense* (98.49%) showed a high degree of similarity, so the obtained strain was determined to belong to the genus *Chryseobacterium* sp. Phylogenetic analysis of the 16S rRNA gene sequences demonstrated that strain BSC2-3 was grouped among *Chryseobacterium* species and closely clustered with *C. gleum* (Figure 1d). Studies on carbofuran degradation using *Chryseobacterium* sp. and *C. gleum* have not been reported thus far. However, *Chryseobacterium* sp. has the ability to degrade solid wastes and pesticides, such as carbendazim [33], flubendiamide [34], organochlorine pesticides [35] and oxyfluorfen [36]. Considering these characteristics, the *Chryseobacterium* sp. BSC2-3 strain is also expected to effectively degrade carbofuran.

### 2.4. Extracellular Changes after Carbofuran Exposure

**Figure 2:**
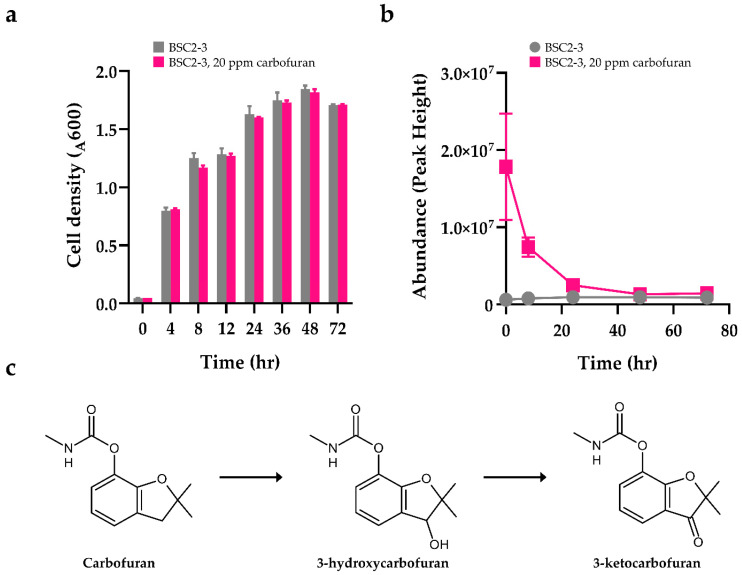
(**a**) Time course of cell growth (optical density at 600 nm, OD_600_) of the BSC2-3 strain during cultivation in R2A liquid medium with 20 ppm carbofuran. Grey bars indicate OD_600_ values from the BSC2-3 strain without carbofuran, while pink bars show cellular growth of the BSC2-3 strain cultured with 20 ppm carbofuran. (**b**) Intracellular carbofuran changes. (**c**) Proposed carbofuran biotransformation pathway in *Chryseobacterium* sp. BSC2-3.

Microbial cultivations were carried out in an R2A medium with (experiment) or without (control) 20 ppm carbofuran. As shown in Figure 2a, BSC2-3 showed moderate growth in the 20 mg/L carbofuran treatment compared with the control. Target compounds (carbofuran and 3-hydroxycarbofuran) were verified based on authentic compounds and identified by matching retention times (RT), accurate mass values (MS1), and MS/MS fragment (MS2) comparisons between standards and samples from cultivation (Figure S1). While a 21% reduction in the level of extracellular carbofuran was observed after 48 h (Figure S2a), the level of intracellular carbofuran was decreased by almost 99% (Figure 2b). The degradation of intracellular carbofuran seemed to accelerate as cell growth progressed. Generally, carbofuran is hydrolysed to 4-hydroxycarbofuran and 5-hydroxycarbofuran when exposed to carbofuran-degrading prokaryotes [37,38,39]. Interestingly, with *Chryseobacterium* sp. BSC2-3, 3-hydroxycarbofuran, which eluted at 3.79 min, was the most abundant metabolite found in the 48 h culture extract (Figure S2b). We further explored whether the BSC2-3 strain has the known carbofuran hydrolases carbaryl hydrolase-encoding gene (*cehA*), the 3-N-methyl carbamate degradation gene (*mcd*) and the carbamate hydrolase gene (*cfdJ*) from *Sphingobium* sp. [31], *Achromobacter* sp. [22] and *Novosphingobium* sp. [40], respectively, by using whole-genome sequencing analyses. However, no homologues of carbofuran hydrolase genes or gene clusters associated with carbofuran hydrolysis were identified in BSC2-3 through sequence blast searching in the coding domain sequences identified through whole-genome sequencing. In contrast, the oxidation of carbofuran and other carbamate pesticides by eukaryotic microbes and insects transforms them into 3-hydroxycarbofuran and 3-ketocarbofuran via a reaction with cytochrome P450 monooxygenases [27]. Moreover, it was suggested that laccase may be involved in carbofuran oxidation [41]. We investigated for cytochrome P450 monooxygenase in the genome of BSC2-3, but fungal P450 monooxygenase [42], which converts carbofuran into 3-ketocarbofuran could not be found in the genome of BSC2-3. Therefore, we propose that the identification of enzymes in *Chryseobacterium* sp. that convert carbofuran to 3-hydroxycarbofuran should be performed in future work. Since various genes coding for intracellular dehydrogenases and monooxygenases (Table 1) exist in *Chryseobacterium* sp., it is suggested that 3-hydroxy carbofuran can be degraded into smaller molecules or converted into several carbofuran derivatives. Based on the identification of extracellular metabolites and whole-genome sequencing results, a predicted pathway for metabolic carbofuran degradation was proposed (Figure 2c). The identified liquid chromatography–mass spectrometry (LC–MS) peaks of 3-ketocarbofuran or further derivatives of 3-hydroxycarbofuran were not completely absent, but they may have been present in very small amounts. This result occurred because the derivatives in the solvent used for extraction and analysis may exist in an unstable form, or the compounds may have already been converted into other compounds related to energy or respiration. The information generated by a single omics study is not always sufficient to understand an entire biological process as complex as microbial bioremediation [43]. However, it is also essential to understand whether the expression of the genes involved in the biodegradation pathways is constitutive, inducible, downregulated or upregulated by the presence of xenobiotics; this information results from the integration of both genomic and transcriptomic technologies. Therefore, further studies are needed to confirm this pathway.

### 2.5. Intracellular Metabolite Profiles of Chryseobacterium sp. BSC2-3

**Figure 3:**
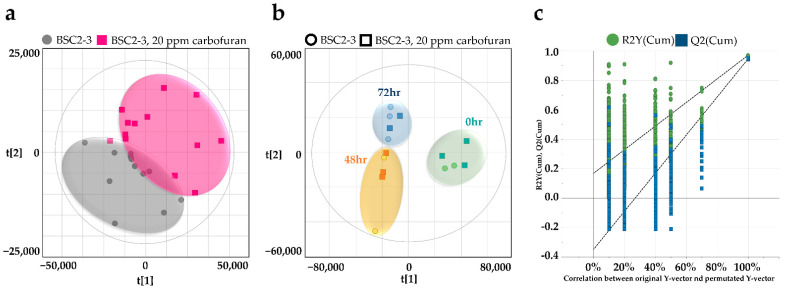
Multivariate analysis of intracellular metabolites from *Chryseobacterium* sp. BSC2-3. (**a**) PLS-DA score scatter plot derived from a total of 299 metabolites between two groups and (**b**) considering the time-dependent effect. (**c**) Validation plot using 999 permutation tests for all samples (R2 was lower than 0.4, and the intercept of Q2 was lower than 0.05).

To understand the changes in the intracellular metabolite distribution depending on the carbofuran treatment of the strain, non-targeted metabolite profiling was performed using LC–MS. A total of 299 intracellular metabolites of the 3450 molecular features were structurally identified in both ionization modes. Metabolomics technology has led biologists to adopt chemometric methods. Especially in biology, metabolomics uses many variables to characterize observations (e.g., samples, treatment and time points). Here, the partial least squares (PLS) method creates interpretable and reliable models capable of processing collinear data structures, and it is an efficient and powerful method for modelling and analysing complex chemical/biological data tables [45]. The classification method, PLS discriminant analysis (PLS-DA), evaluates the reliability of the results by using the resampling bootstrap technique. The advantage of this approach is that the sources of variations in the data are modelled by the latent variable, and then the segregation of the groups can be assessed visually. For this reason, we conducted PLS-DA to obtain unbiased model performance estimates and observed highly different metabolite profiles for the control and experimental groups (Figure 3a). We also observed differences between groups in metabolite changes over time in the PLS-DA model with the double cross-validation scheme (CV-ANOVA P = 4.818 × 10^−5^, Figure 3b). The goodness of fit of the model could be determined by quantifying the R2X (0.731) value of explanatory power and the Q2X (0.663) value of predictive power. Regardless of the carbofuran treatment, the x-axis mainly explained the discrimination of metabolite profiles between 0 h and other cultivation times, and the y-axis could distinguish metabolite changes at 24 h and 72 h of cultivation. The original model validation was performed with 999 random permutation tests (Figure 3c). This result was verified by obtaining the quantitative values of the R2 (0.167) and Q2 (−0.350) intercepts.

### 2.6. Altered Intracellular Metabolism in Response to Carbofuran

**Figure 4:**
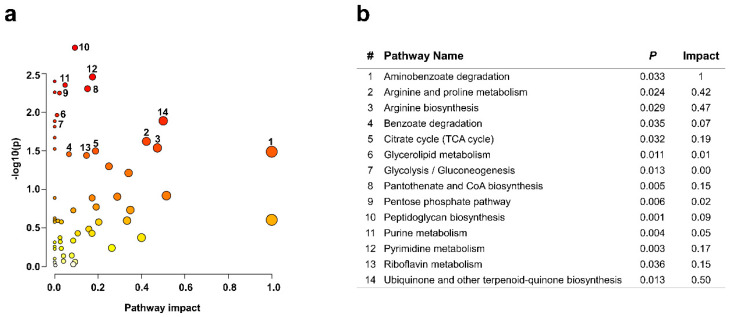
Metabolic pathway analysis using MetaboAnalyst 5.0. Pathway analysis was performed based on the *P. putida* KT2440 KEGG pathway library. (**a**) The *x*-axis represents the pathway impact value from pathway topology analysis, and the *y*-axis represents the −log *p*-value from pathway enrichment analysis. The node colour and radius are based on the *p*-value and pathway impact value, respectively. (**b**) The table represents the *p*-values and impact values of metabolic pathways that were significantly changed in experiments.

Approximately 220 out of the 299 metabolites were classified. These metabolites were subclassified into 120 primary metabolites (15 carbohydrate, 70 amino acid, 20 fatty acid and 15 purine and pyrimidine metabolites) and 100 secondary metabolites related to alkaloids, benzenoids, phenylpropanoids, steroids and other metabolites. When the metabolite changes were observed by focusing on the 72 h culture, it was found that amino acid and nucleoside-related metabolites (amino acid, amino acid derivative and purine and pyrimidine metabolites) were significantly altered compared to the metabolites corresponding to the central carbon and nitrogen metabolic pathways (Table 2). In addition, it was confirmed that benzenoid metabolites were significantly changed in the secondary metabolic pathway in the carbofuran-treated group compared to the control group (Table 2). To investigate the detailed metabolic pathway changes of the altered metabolites, pathway analysis was performed using the Kyoto Encyclopedia of Genes and Genome (KEGG) pathway library of *Pseudomonas putida* KT2440, a well-studied strain for the metabolism of monocyclic aromatic compounds [46] (Figure 4a). As a result, the degradation of aminobenzoate, the biosynthesis of ubiquinone and other terpenoid-quinones biosynthesis, as well as the metabolism of arginine and proline, were the most remarkably different metabolic pathways between the two groups (Figure 4b). Furthermore, similar to the metabolite analytical results, there were insignificant differences in glycolysis and the pentose phosphate pathway between the control and carbofuran-treated groups. Among the central metabolic pathways, the tricarboxylic acid (TCA) cycle was the only metabolic pathway that differed between the two groups. Unexpectedly, remarkable changes in many primary and secondary metabolic pathways associated with arginine were observed in various amino acid metabolic pathways. The metabolism of purine and pyrimidine also showed significant changes (Figure 4b). Aminobenzoate degradation showed the greatest difference in pathway impact analysis, and the metabolic pathways related to the production of 4-hydroxybenzoate as a central metabolite, benzoate degradation and the biosynthesis of ubiquinone and other terpenoid-quinones showed significant differences between the two groups. The high ratio of secondary metabolites among the identified metabolites is likely due to the stress response induced by carbofuran. There is a high probability that useful products will be found among secondary metabolites generated by external stimuli. In the strain *Chryseobacterium antibioticum* sp. nov., plant alkaloid and auxin production-related genes were found [47], and genes related to keratin degradation and oxidative stress resistance [48] were found. In the future, it will be necessary to investigate the mechanism underlying the production of bioactive compounds in microorganisms of the genus *Chryseobacterium*.

### 2.7. Identification of Metabolites Related to Aminobenzoate and Benzoic Acid Catabolism

**Figure 5:**
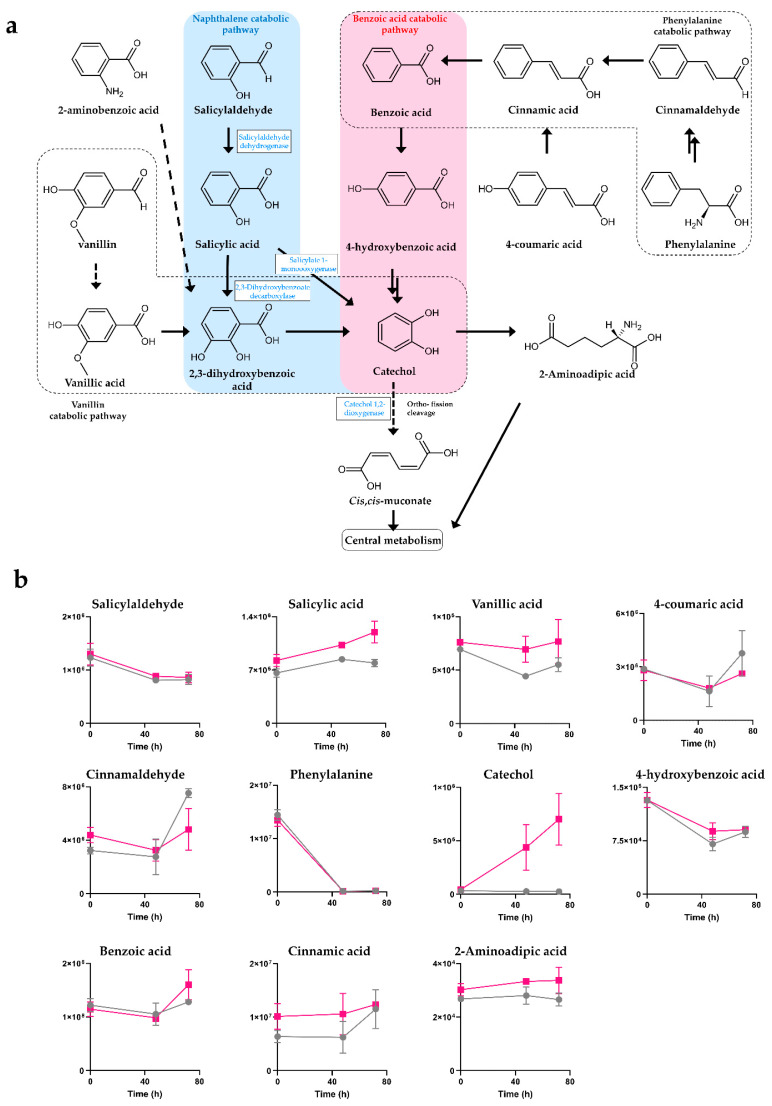
(**a**) Proposed degradation pathways for naphthalene-, benzoic acid-, phenylalanine- and benzene-derived compounds in *Chryseobacterium* sp. BSC2-3. (**b**) Graphs represent time course intracellular changes in salicylic acid, salicylaldehyde, vanillic acid, 4-coumaric acid, cinnamaldehyde, phenylalanine, catechol, 4-hydroxybenzoic acid, benzoic acid, cinnamic acid and 2-aminoadipic acid intensities in the BSC2-3 strain cultured with (pink line) and without (grey line) 20 ppm carbofuran. Chemical structures and pathways were annotated from the KEGG database. Black arrows designate a direct connection between metabolites. Dashed arrows indicate the presence of metabolites that were not detected.

Through non-targeted intracellular metabolite analysis, various benzenoid compounds were identified. A deeper investigation was performed on the aminobenzoate degradation metabolic pathway, which showed the most significant change between the carbofuran-treated group and the control group through metabolic pathway analysis. Among the various monocyclic benzenoids, significant changes in catechol, benzoic acid and salicylic acid were observed in the experimental group compared to the control group. In particular, a 28-fold increase in catechol was observed at 72 h compared to the control group (Table 2). Catechol, benzoic acid and salicylic acid had a strong linkage with the naphthalene and benzoic acid degradation pathways. Changes in the metabolites constituting the phenylalanine degradation pathway seemed to have contributed to the accumulation of catechol, but they do not appear to have a significant effect. Based on the identified benzenoids and their pathways, the benzenoid degradation pathway of *Chryseobacterium* sp. BSC2-3 was proposed via KEGG pathway analysis (Figure 5a). Since BSC2-3 has been shown to have a distinct naphthalene degradation metabolic pathway, it may be capable of degrading carbaryl, which has a similar molecular structure to naphthalene. Since carbofuran is the same N-methylcarbamate class insecticide as carbaryl, there is a possibility that the degradation of carbofuran occurred through the corresponding metabolic pathway, but it was unclear due to the absence of a direct observation of intermediates in the conversion route of carbofuran to catechol. However, it seems clear that the cause of catechol accumulation is carbofuran. Similarly, the change in benzoic acid-related metabolites is considered to be an indirect effect of carbofuran exposure rather than a direct conversion of carbofuran. Since salicylate monooxygenase, an enzyme that converts catechol into *cis*,*cis*-muconate, is present in the genome of BSC2-3 (Table 1), catechol was considered to be used as an energy source through the central metabolic pathway. However, the accumulation of catechol in the carbofuran-treated group remarkably increased in a time-dependent manner. It was assumed that there may be a limiting step triggered by carbofuran or its transformants. In addition, the accumulation of monocyclic compounds, such as 4-hydroxybenzoic acid, 2-aminoadipic acid and vanillic acid, was clearly increased by exposure to carbofuran (Figure 5b).

### 2.8. Identification of Biomolecules for Growth Promotion and Disease Control

During the growth of microorganisms, various primary and secondary metabolites were produced and secreted to the surrounding environment [49]. Various secondary metabolites of soil bacteria fertilize the soil or promote plant growth, and some are used as biological control agents to prevent pests and plant diseases [50,51]. We investigated riboflavin, lumichrome and indole 3-acetic acid (IAA) from the metabolome to verify the BSC2-3 strain as an effective microorganism in the agriculture field. Since guanosine triphosphate (GTP) is a key precursor for riboflavin synthesis [52] and the BSC2-3 strain has its own riboflavin biosynthetic pathway (Table 1), a dramatic change in riboflavin synthesis was predicted for the carbofuran-treated group. Although no association was found between carbofuran and riboflavin accumulation (Figure S3a), the extracellular riboflavin content was increased in a time-dependent manner (Figure S3b). In contrast, the content of lumichrome, a degradation product of riboflavin, was increased in a time-dependent manner both intra- and extracellularly (Figure S3c,d). Moreover, the production of lumichrome in the carbofuran-treated group was increased by 63.9% compared to that in the control group after 72 h of cultivation (Figure S3c). Since riboflavin can control plant diseases [53] and lumichrome has been reported to support plant growth [54,55,56], the BSC2-3 strain is expected to be a beneficial soil bacterium. A gene coding for indole-3-acetic acid (IAA) amido synthetase in the genome of *Chryseobacterium* BSC2-3 was identified (Table 1). In plants, the activation of IAA amido synthetase promotes salicylate production [57]. A similar phenomenon was observed in the BSC2-3 strain, which may be one of the reasons for the intracellular accumulation of salicylic acid and catechol. IAA is one of the most active auxins and plays a role as a plant growth regulator. Several microorganisms, including plant growth-promoting rhizobacteria (PGPR), have been isolated to produce IAA. The genus *Chryseobacterium* has been regarded as a plant growth-promoting bacterium source in various studies [58,59]. A previous study revealed that *C. gleum* sp. produced 61.18 ± 0.5 µg/mL IAA during the 24 h of its cultivation [60]. Our data showed that extracellular secretion of IAA was increased in a time-dependent manner (Figure S4a), while intracellular IAA production decreased (Figure S4b). Correlated changes in extracellular and intracellular IAA and riboflavin levels appear to be unique features of the strain itself rather than an effect of carbofuran. More research is needed on the mechanism and action of plant growth and disease control substances of *Chryseobacterium* BSC2-3. Although carbofuran exposure did not affect riboflavin and IAA production, it is very meaningful to be able to measure simultaneous extracellular and intracellular disease resistance inducers and plant growth regulators through non-targeted metabolomics. Considering the observations above, the complete metabolism of compounds consisting of a fused pair of benzene rings, such as naphthalene and carbaryl, would be achieved in the BSC2-3 strain with plant growth-supporting ability.

## 3. Materials and Methods

### 3.1. Chemicals

Carbofuran (purity, 98%) and 3-hydroxycarbofuran (purity, 99%) were purchased from Sigma Aldrich (Milwaukee, WI, USA). All solvents used for QuEChERS extraction and LC–MS operation were of LC–MS grade and purchased from Fisher Scientific (Pittsburgh, PA, USA). All other chemicals used were of reagent grade unless otherwise noted.

### 3.2. Enrichment and Isolation of Carbofuran-Degrading Strains

Contaminated soil samples were collected from farmland in Boseong-gun, Korea (N 34°52′08.1″ and E 127°19′11.6″). The major crops in this area are beans, corn and chilli peppers. To prepare fine-grained soil, the collected soil was sifted twice by using a sieve with a 2 mm pore size (2.0 mm Test Sieve; ChungGye sieve co., Ltd., Seoul, Korea). MSM was prepared, and contained the following components: 2 g/L KH_2_PO_4_, 7.5 g/L K_2_HPO_4_, 0.1 g/L MgCl_2_, 0.5 g/L NaCl, and 1 mL/L trace element solution. The trace element solution was composed of the following compounds (mg/L): (CH_3_COO)_2_Cu·H_2_O, 10; CoCl_2_·6H_2_O, 3; EDTA, 500; FeCl_2_·6H_2_O, 20; (NH_4_)_6_Mo_7_O_24_·4H_2_O, 20 and ZnCl_2_, 30. The enrichment culture was started by adding 5 g of the prepared soil and 500 μL of a 10,000 ppm carbofuran stock solution to 100 mL of sterilized MSM in 250 mL sterilized Erlenmeyer flasks. After 2 weeks of inoculation, 5 mL of culture medium was transferred to the fresh MSM with 100 ppm carbofuran. This procedure was repeated three times. The shaking incubator was kept dark throughout the culture process. The final enriched medium was diluted in sterile water (3M, USA) and spread on an R2A-agar medium to isolate a single colony.

### 3.3. Identification of Carbofuran-Degrading Bacteria

The 16S rRNA sequence analysis of the carbofuran-degrading strain was performed by Macrogen (Seoul, Korea). A phylogenetic tree was constructed using the neighbour-joining method based on nucleotide sequences of the isolates with reference strain sequences from the EzBioCloud database (https://www.ezbiocloud.net/, accessed date: 26 August 2021) of ChunLab Inc. (Seoul, Korea) using Mega-X software (version 10.2.1) with 1000 bootstrap replicates.

### 3.4. Confirmation of Carbofuran Degradation Using TLC Analysis

#### 3.4.1. Culture Conditions

The BSC2-3 strain was cultivated in 10 mL of R2A medium at 30 °C for 12 h to 14 h. Individual cultures were conducted in 100 mL of R2A medium containing 50 mg/L carbofuran. The culture was carried out at 30 °C and 100 rpm. Culture broth was intermittently collected every 24 h for QuEChERS extraction and TLC analysis.

#### 3.4.2. QuEChERS Extraction

Samples were extracted with acetonitrile (ACN) according to the QuEChERS-EN method. Ten millilitres of culture sample were mixed with 10 mL of ACN in a 50 mL conical tube and vortexed for 30 s. A QuEChERS powder mix (4 g of magnesium sulfate, 1 g of sodium chloride, 4 g of trisodium citrate dihydrate and 0.5 g of disodium hydrogen citrate sesquihydrate) was added to the conical tube and vortexed again for 30 s. Centrifugation was performed for 10 min in a high-speed centrifuge at 10,000 rpm. After centrifugation, 5 mL of the supernatant (organic phase) was filtered using a 10 mL syringe and a 0.2 μm-pore size PTFE filter (Advantec, Tokyo, Japan). The filtered culture extract was stored at −70 °C for further analysis.

#### 3.4.3. TLC Analysis

TLC silica plates (aluminium HPTLC silica gel 60 F_254_ plates, Merck, Darmstadt, Germany) were cut into 5 cm × 5 cm or 5 cm × 10 cm pieces according to the purpose of the experiment. To measure the minimum detectable range, 5 μL of various concentrations (50–10,000 ppm) of carbofuran solution was spotted on a TLC plate and developed in 5:5 ethyl acetate/hexane. After the TLC plate was developed, it was allowed to dry for 3–5 min, and then spots were detected under UV light at 254 nm. For the qualitative evaluation of carbofuran using TLC, the concentration of carbofuran in the sample was 5000 ppm. Two millilitres of the culture extract were volatilized using a centrifugal vacuum concentrator (HyperVac, GYROZEN, Gimpo, Korea) connected to a cooling trap device (HyperCool, GYROZEN, Gimpo, Korea) at 37 °C until the solvent was completely evaporated. The residue was dissolved in 5 mL of acetone to make a 50-fold concentrated sample. Five microlitres of concentrated extracts were spotted on the plate with 5000 ppm carbofuran in acetone as a reference.

### 3.5. Extra/Intracellular Metabolite Profiling of the BSC2-3 Strain

#### 3.5.1. Culture Conditions

For extra/intracellular metabolite analysis, the *Chryseobacterium* BSC2-3 strain was cultured in 10 mL of R2A medium and incubated at 30 °C and 100 rpm for 12 h. Seed-cultured cells were inoculated into 100 mL of fresh R2A medium supplemented with 20 ppm carbofuran until an OD_600_ of 0.5 was reached. The main cultures were operated at 30 °C and 100 rpm. The cultures were conducted in triplicate to measure carbofuran degradation and the intracellular metabolite profiles in a time-dependent manner. The pH values were calculated using an ORION STAR A211 pH meter (Thermo Scientific, Waltham, MA, USA) equipped with an Orion 8115BNUWP ROSS Ultra Electrode.

#### 3.5.2. Preparation of Samples for Metabolite Profiling

The culture broth was centrifuged at 6000 rpm and 4 °C for 5 min, and 20 µL of each culture supernatant was collected and stored at −70 °C. The supernatants were thawed and evaporated at 30 °C using a vacuum rotary evaporator. The pellet was reconstituted in 200 μL of 70% ACN. Cells were collected to reach an OD_600_ of 0.5 via centrifugation at 10,000 rpm and 4 °C for 2 min. The cell pellets were washed twice with 10 mL of ice-cold 70% methanol (MeOH), lyophilized for 24 h, and stored at −70 °C for further use. The lyophilized cells were prepared with a homogenizer (Precellys 24; Bertin Technologies, Montigny-le-Bretonneux, France). The ground sample was mixed with 750 μL of extraction solvent (DW:MeOH = 5:9) and centrifuged for 5 min at 13,000 rpm and 4 °C. Then, 650 μL of the supernatant was transferred to a new e-tube and evaporated at 40 °C using a vacuum rotary evaporator. The pellets containing the metabolites extracted from the bacterial cells were stored at −70 °C until LC–MS analysis.

#### 3.5.3. LC–MS Analysis

The analysis of extra- and intracellular metabolites was performed using an UPLC (Ultimate^TM^ 3000 RSLC system, Dionex Inc., Amsterdam, The Netherlands) with a QE Orbitrap MS (Thermo Fisher Scientific, Waltham, MA, USA) system equipped with an Xbridge amide column (4.6 × 100 mm, 3.5 µm, Waters, Milford, MA, USA). The following gradient was applied using 5 mM ammonium acetate (mobile phase A) and 100% ACN (mobile phase B) at a column flow of 0.30 mL/min and temperature of 30 °C: 0–3 min, 90 to 30% B; 3–12 min, 30 to 2%; 12.0–17.0 min, 2% B; 17.0–17.1 min, 2 to 90% B; and 17.1–23 min, 90% B, for re-equilibration. A 5 µL aliquot of the sample extract was injected into an injector port of an autosampler maintained at 5 °C during analysis. All analyses were performed in both ionization modes (negative and positive) based on the data-dependent MS/MS mode (*m*/*z* range: 60–900). The quality control (QC) samples obtained by mixing all intracellular extracts were injected every 10 runs [61]. The coefficient of variation (CV) and dilution factor of the QC sample were utilized to ensure analyte reproducibility and stability.

### 3.6. Metabolite Data Processing

Carbofuran and 3-hydroxycarbofuran screening and quantification were performed using the Quan method by selecting compounds from the compound database (CDB) function of TraceFinder 4.1 (Thermo Fisher Scientific, Waltham, MA, USA). The CDB was built of accurate masses and RTs based on individual standard compound spectra. The method was developed with the following parameters: RT window: 15 s; mass accuracy: 5 ppm. Non-targeted metabolite analysis was performed using an MS-DIAL version 4.60 (RIKEN, Kanagawa, Japan). First, all sample data, including blank and QC data (vendor format: .raw), were converted to the ABF format using Abf Converter version 4.0.0 (Reifycs Inc., Tokyo, Japan). Then, peak detection and chromatogram deconvolution were conducted using the MS-DIAL with data-dependent methods within mass tolerances of 0.05 (MS1) and 0.075 (MS2) in each ionization mode. We matched the deconvoluted spectra using built-in library mass spectra based on the mass spectral similarity within 80% of the cut-off threshold [62,63]. Finally, the aligned results after peak alignment, filtering and gap filling included values in which the average peak height of the samples was more than five-fold that of the blank.

### 3.7. Statistical Analysis

The data were expressed as the mean ± standard error of the mean (SEM) and were analysed using GraphPad Prism, version 9.0.0 (GraphPad Software, San Diego, CA, USA). Univariate statistics were performed using Student’s *t* test, and multivariate statistics for multiple groups were accomplished by PLS-DA and variable importance in projection (VIP) using SIMCA Version 16.0.1 (Umetrics, Umeå, Sweden). Additionally, pathway analysis was conducted using MetaboAnalyst 5.0 (https://www.metaboanalyst.ca/, accessed date: 9 August 2021) to determine the significantly altered metabolism caused by the carbofuran treatment. The data were transformed using sum normalization and autoscaling, global test algorithms were used as parameters for specifying pathway analysis and topological analysis was performed using relative betweenness centrality.

### 3.8. Whole-Genome Sequencing

The BSC2-3 strain was cultivated in 500 mL of tryptic soy broth at 30 °C and 100 rpm for 16 h. The cells were harvested by centrifugation at 5000 rpm and 4 °C. After centrifugation, the cell supernatant was discarded, and the cell pellet was kept at 4 °C before analysis. Whole-genome sequencing was commercially performed by ChunLab, Inc. (Seoul, South Korea), using an Illumina MiSeq (Illumina, San Diego, CA, USA) sequencing system. Genome information of *Chryseobacterium* sp. BSC2-3 is available in the GenBank database (accession number: JAJQWT000000000). The general features of the genome of the BSC2-3 strain are summarized in Table S1.

## 4. Conclusions

The results described in this report showed the intracellular metabolomic alteration of carbofuran through non-targeted metabolomics. We developed methods for providing macroscopic observations of extra- and intracellular changes. This is the first report of the microbial oxidation of carbofuran and the generation of unique metabolites of carbofuran by the genus *Chryseobacterium*. Based on the results reported in this study, tentative pathways for the catabolism of carbofuran and benzenoid compounds were proposed. The potential of BSC2-3 as a useful agricultural microorganism was confirmed through the analyses of riboflavin, lumichrome and IAA production and its related genes. Considering the results in this study, non-targeted metabolomics could be a potent strategy to identify intracellular changes after exposure to toxic materials, including pesticides and benzoic and phenolic compounds.

## Figures and Tables

**Table 1:** Genes involved in carbofuran, naphthalene, aminobenzoate degradation and indole-3-acetic acid (IAA) production by *Chryseobacterium* sp. BSC2-3 and their associated pathways sorted with KEGG orthology (KO) terms.

Pathway Name	Definition	KO ID ^1^
Aromatic hydrocarbons catabolism	Putative ring-cleaving dioxygenase *MhqO*	K15975
	Putative ring-cleaving dioxygenase *MhqA*	K06999
	Putative salicylate 1-monooxygenase	K00480
Riboflavin synthesis	Riboflavin synthase	K00793
	Riboflavin kinase	K11753
	GTP cyclohydrolase II	K01497
	3,4-dihydroxy 2-butanone 4-phosphate synthase	K02858
	3,4-dihydroxy 2-butanone 4-phosphate synthase and	K14652
	GTP cyclohydrolase II	
	5-amino-6-(5-phosphoribosylamino) uracil reductase	K11752
	6,7-dimethyl-8-ribityllumazine synthase	K00794
Plant hormone signal transduction	Auxin responsive *GH3* gene family	K14487

^1^ In accordance with KEGG (Kyoto Encyclopedia of Genes and Genomes database) [44].

**Table 2:** Classification table of metabolites significantly changed in the carbofuran-treated group.

Class	Metabolites	*p*-Value	Fold Change
Carbohydrates and carbohydrate	Gentiobiose	0.014	2.10
conjugates	Gluconate	0.010	2.38
	Glucosamine	0.016	1.76
	Glucose	0.002	1.46
	Glyceric acid	0.019	1.61
	Maltotriose	0.029	3.12
	Threonic acid	0.048	1.76
Amino acids and derivatives	Arginine	0.010	1.85
	Betaine	0.044	1.56
	Hydroxyproline	0.003	2.80
	Lysine	0.040	1.68
	N-Epsilon-acetyllysine	0.004	2.92
	N-Formyl-L-methionine	0.010	2.42
	O-Acetyl-L-homoserine	0.011	2.11
	Pipecolic acid	0.026	2.34
	Saccharopine	0.004	5.07
	Trans-4-hydroxy-L-proline	0.048	1.49
Purines and pyrimidines	Adenine	0.001	1.78
(+derivatives)	Cordycepin	0.041	2.27
	Cytosine	0.005	1.65
	Isonicotinic acid	0.007	2.20
	Norharman	0.049	3.12
	Thymidine	0.014	1.75
Benzenoids	4-hydroxybenzoate	0.048	1.52
	Catechol	0.033	27.95
	Salicylic acid	0.047	1.51

## Data Availability

The data presented in this study are available on request from the corresponding author. The data are not publicly available due to further analysis ongoing.

## Supplementary Materials

The following are available online at https://www.mdpi.com/article/10.3390/metabo12030219/s1, Figure S1: Extracted ion chromatograms (EICs) of the target compounds carbofuran (a) and 3-hydroxycarbofuran (b) in positive ionization mode. The mirror plot represents the concordance of MS/MS fragments of standard (up-blue) and library (down-red) in carbofuran (c) and 3-hydroxycarbofuran (d). Mass fragment spectral results of carbofuran (e) and 3-hydroxycarbofuran (f) in the real samples. Figure S2: The graph shows the changes in the relative abundance of carbofuran (a) and 3-hydroxycarbofuran (b) in the supernatant in a time-dependent manner. Figure S3: Alterations in the relative abundance of intracellular (a), (c) and extracellular (b), (d) riboflavin (a), (b) and lumichrome (c), (d) in a time-dependent manner. Figure S4: Time course extracellular (a) and intracellular (b) changes in indole-3-acetic acid content. Table S1: General features of *Chryseobacterium* sp. BSC2-3 genome.
