# Supplementary material for: Screening of Carbofuran-Degrading Bacteria Chryseobacterium sp. BSC2-3 and Unveiling the Change in Metabolome during Carbofuran Degradation
Source: Metabolites. 2022 Mar 1;12(3):219. doi: 10.3390/metabo12030219 (PMC8950912; doi:10.3390/metabo12030219)
Supplement: Supplementary file 1 [file metabolites-12-00219-s001.zip › metabolites-1495789-supplementary.pdf]

Screening of Carbofuran-Degrading Bacteria *Chryseobacterium* sp. BSC2-3 and Unveiling the Change in Metabolome during Carbofuran Degradation

Haeseong Park [1], Sun Il Seo [1], Ji-Hwan Lim [1], Jaekyeong Song [2], Joo-Hyun Seo [3,*] and Pyoung Il Kim [1,*]

[1] Center for Industrialization of Agricultural and Livestock Microorganisms, 241 Cheomdangwahak-ro, Jeongeup-si, Jeollabuk-do 56212, Korea; haeseong@cialm.or.kr (H.P.); siseo@cialm.or.kr (S.I.S.); ljh0408@cialm.or.kr (J.-H.L.)

[2] Division of Agricultural Microbiology, National Academy of Agricultural Science, Rural Development Administration, Jeonju 56050, Korea; mgjksong@korea.kr

[3] Department of Bio and Fermentation Convergence Technology, Kookmin University, Seoul 02707, Korea

* Correspondence: joohyunseo@kookmin.ac.kr (J.-H.S.); pikim30@cialm.or.kr (P.I.K.); Tel.: +82-63-536-6001 (P.I.K.)

Table S1. General features of the *Chryseobacterium* sp. BSC2-3 genome.

Characteristic	Genome	Characteristic	Genome
Genome size (bp)	4786171	Mean of CDS length (bp)	980.5
GC content (%)	36.8	Median of CDS length (bp)	804
Number of contigs	14	Mean length of intergenic region	135.2
Number of CDSs	4312	Number of rRNA genes	6
N50 (bp)	503816	Number of tRNA genes	80

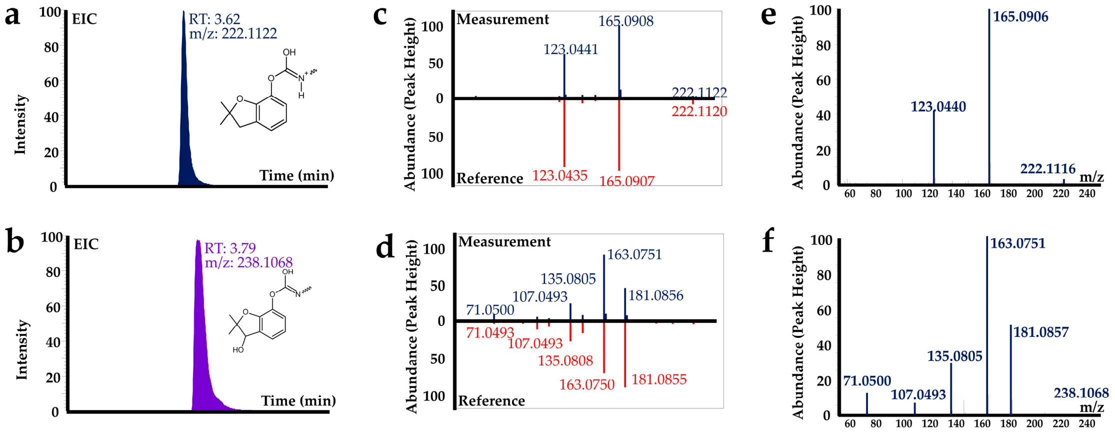

Figure S1. Extracted ion chromatograms (EICs) of the target compounds carbofuran (**a**) and 3-hydroxycarbofuran (**b**) in positive ionization mode. The mirror plot represents the concordance of MS/MS fragments of standard (up-blue) and library (down-red) in carbofuran (**c**) and 3-hydroxycarbofuran (**d**). Mass fragment spectral results of carbofuran (**e**) and 3-hydroxycarbofuran (**f**) in the real samples

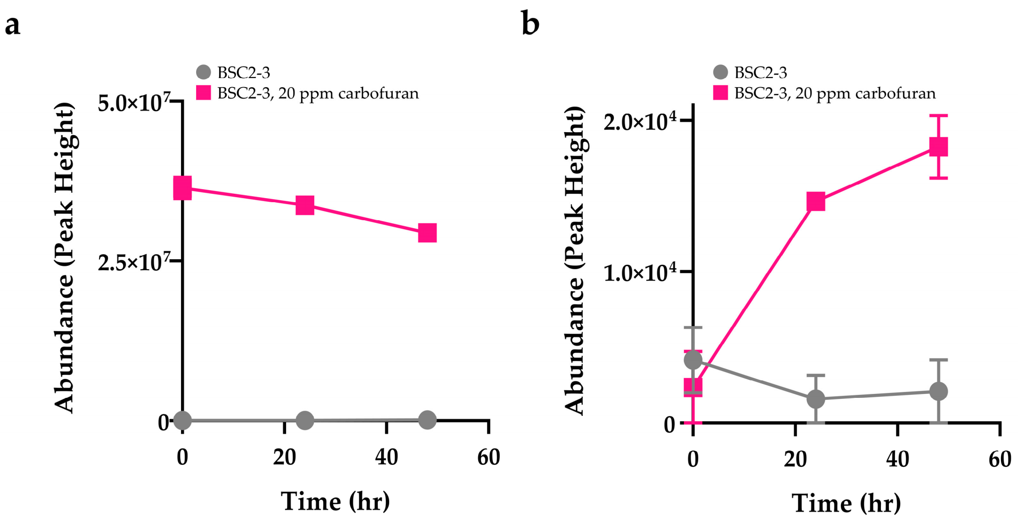

Figure S2. The graph shows the changes in the relative abundance of carbofuran (**a**) and 3-hydroxycarbofuran (**b**) in the supernatant in a time-dependent manner

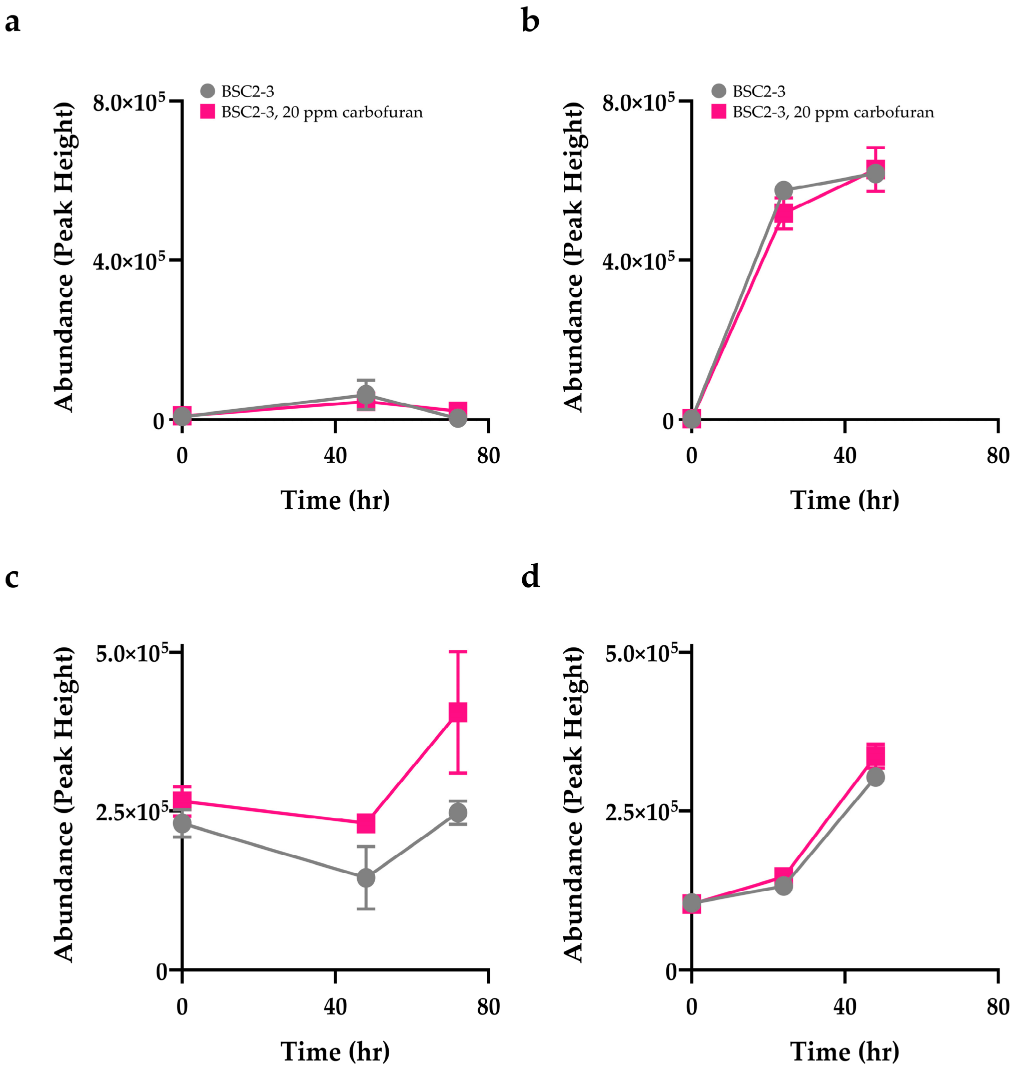

Figure S3. Alterations in the relative abundance of intracellular (**a**), (**c**) and extracellular (**b**), (**d**) riboflavin (**a**), (**b**) and lumichrome (**c**), (**d**) in a time-dependent manner

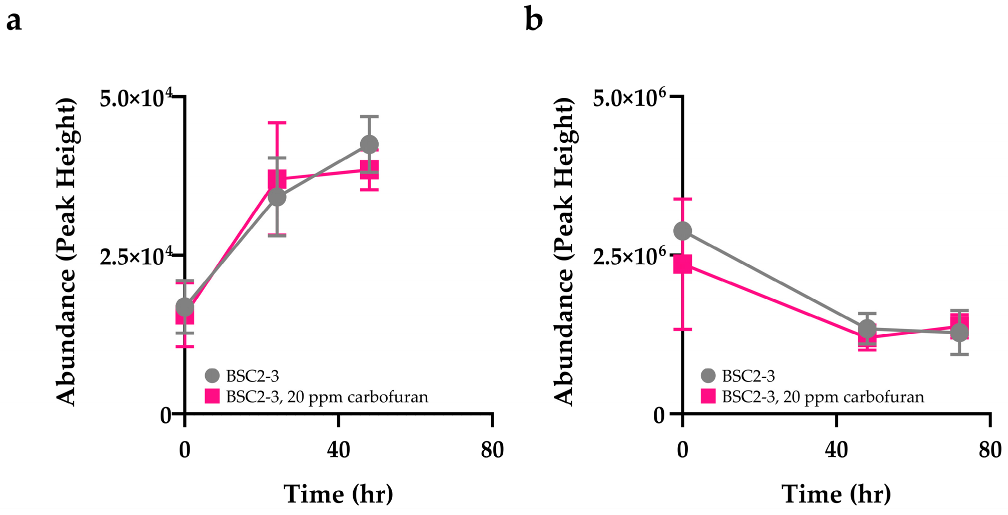

Figure S4. Time-course extracellular (a) and intracellular (b) changes in indole-3-acetic acid content
